# Precipitation and Interannual Variability Shape the Phenology and Abundance of the Endangered Butterfly *Baronia brevicornis*

**DOI:** 10.1007/s13744-025-01325-y

**Published:** 2025-12-23

**Authors:** Yesenia Alvarado-Campos, Gloria Ruiz-Guzmán, Carlos A. Anaya Merchant, Elaine M. Méndez Muñiz, Jorge Contreras-Garduño

**Affiliations:** 1 Escuela Nacional de Estudios Superiores, Unidad Morelia, UNAM, Morelia, Michoacán México; 2 Posgrado en Ciencias Biológicas, UNAM, Coyoacán, Ciudad de México México

**Keywords:** Panchronic species, Diapause, Polymorphism, Climate change, Endemic, Plasticity, Precipitation

## Abstract

Insects in tropical regions are subject to pronounced seasonal fluctuations in rainfall and temperature. A notable example is *Baronia brevicornis*, a panchronic butterfly species endemic to Mexico. Its reproductive season begins with the onset of the rainy period, which triggers the emergence of adults. Males exhibit four distinct color morphs, while females display three. To assess adult abundance, sex ratios, morph proportions, and wing size variation, we monitored a population of *B. brevicornis* during mating seasons (2015, 2017, 2018, 2019, 2021, 2023, and 2024). In addition, we experimentally investigated the effects of prolonged diapause and pupal survival. Our findings indicate a male-biased sex ratio. Morph frequencies and wing sizes in both sexes exhibited temporal variation. Adults emerged following one to two years of pupation when no water was provided during the first year. However, the proportion of individuals successfully emerging after two years was significantly lower than in those emerging after a single year. Our model suggests that accumulated precipitation, rather than temperature, plays a critical role in the probability of detecting adult butterflies. The optimal threshold (~ 70–150 mm) may be an adaptive strategy to cope with unpredictable rainfall, enabling individuals to postpone emergence when precipitation levels are insufficient to sustain host plants and nectar resources. Nevertheless, the potential fitness costs associated with prolonged pupation must be considered.

**Supplementary Information:**

The online version contains supplementary material available at 10.1007/s13744-025-01325-y.

## Introduction

Panchronic species are those that preserve relatively unchanged ancient phenotypes and are the vestiges of extinct lineages (Grassé 2013; Grandcolas et al. 2014). These species typically inhabit geographically isolated and ecologically restricted areas (Habel et al. 2010), persisting due to their ability to exploit specific ecological niches (Grassé 2013). A well-known example is the tuatara (*Sphenodon punctatus*), a reptile endemic to New Zealand whose lineage dates back approximately 250 million years (Gemmell et al. 2020). This species exhibits behavioral thermoregulation, enabling activity across a broad temperature range (5–35 °C), including extremes unsuitable for most reptiles (Corkery et al. 2014). Given their evolutionary significance and ecological specificity, panchronic species demand careful consideration in conservation planning. Global climate change poses a serious threat to these taxa, as the climatic niches that have historically served as refugia may contract, potentially leading to population declines (Habel et al. 2010; Rödder et al. 2010). For instance, the relict conifer *Abies koreana*, endemic to alpine and subalpine zones, is experiencing habitat contraction and increasing vulnerability to drought (Koo et al. 2017). Likewise, panchronic species in Mediterranean ecosystems have shown signs of water stress attributed to ongoing climate change and, to a lesser extent, natural drought cycles (Garfi & Buord 2012).

Climate change significantly alters global precipitation patterns, generally increasing rainfall in humid regions while reducing precipitation in arid and subtropical zones (Trenberth 2011). These shifts disrupt seasonal precipitation cycles, with cascading effects on reproductive success and phenological events (Forrest 2016). In many insect species, precipitation plays a critical role in regulating emergence timing, which in turn influences the number of generations per year and the development of life stages such as diapause, factors that collectively shape population dynamics (Lindberg & Bengtsson 2005; Oliver et al. 2015). However, the specific impacts of changing precipitation and temperature regimes on the population dynamics of panchronic species remain poorly understood. This gap underscores the urgent need to establish long-term population monitoring programs. Such efforts are essential for assessing the vulnerability of these evolutionarily distinct species and for developing evidence-based conservation strategies. For tropical species, integrating both biological factors and climate variability is essential to understanding how precipitation patterns affect phenology and population dynamics (Henry et al. 2022).

The panchronic species *Baronia brevicornis* (ESM_Figure 1), is one of the least-studied members of the family Papilionidae, is endemic to Mexico, and represents the oldest extant butterfly lineage globally (Eisner 2003; Legal et al. 2015; Espeland et al. 2018; Kawahara et al. 2019). Its distribution is highly restricted, largely due to its stringent biotic and abiotic requirements (Legal et al. 2015; Galicia-Mendoza et al. 2021). The species’ survival is closely tied to the availability of specific host plants *Acacia cochliacantha* (syn. *Vachellia cochliacantha*; Fabaceae) for larvae (Vázquez and Pérez 1961; 1966; Covarrubias-Camarillo et al. 2016), and *Bursera copallifera* (Burseraceae), *Lysiloma divaricatum* (Fabaceae), and *Tournefortia densiflora* (Boraginaceae) for adult nectar feeding (Galicia-Mendoza et al. 2021). While it has been hypothesized that temperature and accumulated precipitation are key factors regulating pupal development and adult emergence (Vázquez and Pérez 1961; Galicia-Mendoza et al. 2021), the specific thresholds of these environmental variables that influence the likelihood of emergence and flight remain largely unknown.

*Baronia brevicornis* exhibits both sexual dimorphism (Collins & Morris 1985) and sexual polymorphism (Galicia-Mendoza et al. 2021). In this species, there are four distinct male color morphs: light brown, dark brown, yellow, and brown with orange spots. Females exhibit three morphs: a light brown morph similar to that of males; a melanic morph characterized by predominantly dark brown wings with white spots; and an orange morph (Galicia-Mendoza et al. 2021). The pupal stage of this butterfly is characterized by an extended period of underground diapause, during which individuals remain buried for most of the year until environmental conditions become favorable for emergence. Diapause is an adaptive strategy that enables organisms to withstand prolonged periods of unfavorable environmental conditions, with its initiation regulated by external cues. During this phase, metabolic activity is reduced, although development continues at a slow rate (Behrens 1984; Tauber et al. 1986). In some species, diapause duration can extend over multiple seasons depending on the availability of critical resources, which are often restricted to a specific time of year (Powell 2001). Both pupation and adult emergence are closely tied to the onset of the rainy season. The first substantial rainfall events soften the soil, facilitating the final stages of pupal development, emergence, and subsequent mating (Vázquez and Pérez 1961). Thus, accumulated precipitation appears to be the primary environmental cue that terminates diapause and initiates adult emergence (Galicia-Mendoza et al. 2021).

The ecological specificity of *B. brevicornis* raises important questions regarding the stability of its morphological traits over time. Do male and female color morphs and body sizes remain consistent across years? How do accumulated precipitation and temperature influence the timing of adult emergence? Prolonged diapause exceeding one season has been documented in several insect species. For example, the moth *Rothschildia jorulla* can emerge after eight years of dormancy (Lees 1955), and *Prodoxus inversus* has been reported to remain in diapause for up to 30 years (Powell 2001). The diapause duration in *B. brevicornis* may also be extended under unfavorable environmental conditions. During annual monitoring, adult emergence was nearly absent in 2018, with only two individuals recorded. Nonetheless, adult butterflies were observed in flight in 2019, despite the apparent absence of egg production in the preceding year. Given the species’ annual or biennial life cycle, we hypothesize that *B. brevicornis* is capable of entering prolonged diapause spanning more than one year and that fluctuations in its population dynamics may be driven primarily by variation in precipitation.

## Materials and Methods

### Study Site and Sampling Method

Butterflies were sampled in Morelos, Mexico. To support the conservation of this species, the specific location of the population is not disclosed in this publication. It may be provided to researchers upon request, along with information regarding the necessary permits in coordination with the relevant authorities. The study area has a warm subhumid climate classified as (Awo’’(w)(i’)g) (García 1964; Pérez-Ruız 1977), the driest type within the subhumid category, with summer rainfall and a pronounced dry season in winter (< 5% of annual precipitation). Based on data from 1990 to 2024, the mean annual precipitation is 823 mm (range: 493–1303 mm), and the mean annual maximum temperature is 34 ºC (range 31.7–37.2 ºC) (CONAGUA 2019; see ESM_Figure 2). Monthly accumulated precipitation and monthly mean maximum temperature for the study years (2015, 2017, 2018, 2019, 2021, 2023, and 2024) are shown in ESM_Figure 3.

Sampling was carried out between May and July (the flight period of *B. brevicornis*) in the years 2015, 2017, 2018, 2019, 2021, and 2024, coinciding with the species’ emergence and reproductive season (Galicia-Mendoza et al. 2021). In 2015, only abundance data were collected, while wing size measurements were only available for 2017, 2019 and 2021. Notably, only two adult individuals were recorded in 2018; however, daily surveys were conducted with the same sampling effort as in other years. For this reason, 2018 was included in the Generalized Additive Model (GAM) analyses to account for the full spectrum of interannual variation, including near-absence events, but excluded from the N-mixture model due to the lack of repeated counts required to estimate detectability and abundance (Royle 2004). Butterflies were captured using entomological nets between 10:00 and 14:00 h, which corresponds to the peak activity period of adults (Galicia-Mendoza et al. 2021). The sampling area consisted of a 150 × 85 m quadrant, with five collectors positioned throughout the site (see also Galicia-Mendoza et al. 2021). Each captured individual was measured (right forewing length from base to apex), sexed, and classified by morph. Marking was performed using indelible Sharpie® markers on the forepart of the right forewing, following a sequential numbering system: numbers up to 99 were used initially, with a dot added for each additional hundred and a line for every thousand (Galicia-Mendoza et al. 2021). All individuals were released immediately after marking.

### Abundance Estimation using N-mixture Models

In order to estimate population abundance while accounting for imperfect detection, we employed N-mixture models as proposed by Royle (2004), utilizing the function pcount() from the *unmarked* package in R. These models are particularly well-suited for populations characterized by low recapture rates, such as *B. brevicornis* (Galicia-Mendoza et al. 2021). Due to the high variability observed in annual counts, a Negative Binomial (NB) distribution was used instead of a Poisson distribution, as it includes a dispersion parameter allowing variance to exceed the mean (Knape et al. 2018). Model comparisons were performed treating ‘year’ as either a continuous or categorical covariate, under both NB and Poisson distributions. The best-supported model (lowest AIC = 3306.90) treated 'year' as a continuous covariate under a NB distribution, using data from 2015, 2017, 2019, 2021, and 2024. The response variable was the total number of butterflies per survey. Our analysis included covariates such as year, proportion of males, maximum temperature, and accumulated precipitation in the month preceding the first capture of each season. The proportion of males was included to test for potential sex-based effects on abundance.

### Climate Data

Daily precipitation and temperature data were obtained from the Jojutla meteorological station (code JOJMR; 18º 37′ 02″ N; 99º 11′ 02″ W; 915 m.a.s.l.), part of the National Network of Meteorological Stations of CONAGUA (CONAGUA, 2019). Accumulated precipitation and mean maximum temperature for each study year were calculated for the month prior to the first observed flight of adults to evaluate their influence on emergence and detectability.

### Diapause Experiment

To investigate the influence of diapause duration on survival, 21 larvae of *B. brevicornis* were collected in July 2019 and maintained in Falcon tubes containing peat moss within a moisture-free incubator set at 27 °C. The larvae developed into pupae within the first week post-collection. In July 2020, nine pupae were selected and subjected to daily watering (5 mL) to simulate rainy conditions, while the remaining pupae were kept dry until July 2021, when they were watered under the same conditions. Adult emergence (as the proportion of pupae that successfully metamorphosed into adults) was recorded in July 2020 and July 2021.

### Statistical Analyses

All analyses were performed in R version 4.3.3 (R Core Team 2024). Sex ratios across years and differences in emergence between one and two years of diapause were tested with Pearson’s chi-square tests. When significant, pairwise comparisons were performed using proportion tests with Bonferroni correction. Changes in morph frequencies across years were assessed separately for males and females using Fisher’s exact test with Monte Carlo simulations (10,000 replicates) to approximate p-values, as recommended when expected frequencies are low (Hope 1968). Generalized Linear Models (GLMs) were applied to assess the effect of morph on wing size for each season. Finally, Generalized Additive Models (GAMs) with a Negative Binomial distribution were used to evaluate linear and non-linear effects of year, maximum temperature, and accumulated precipitation on butterfly captures.

## Results

### Population Structure: Sex Ratio, Frequency of the Morphs, Wing Size, and Abundance

**Fig. 1:**
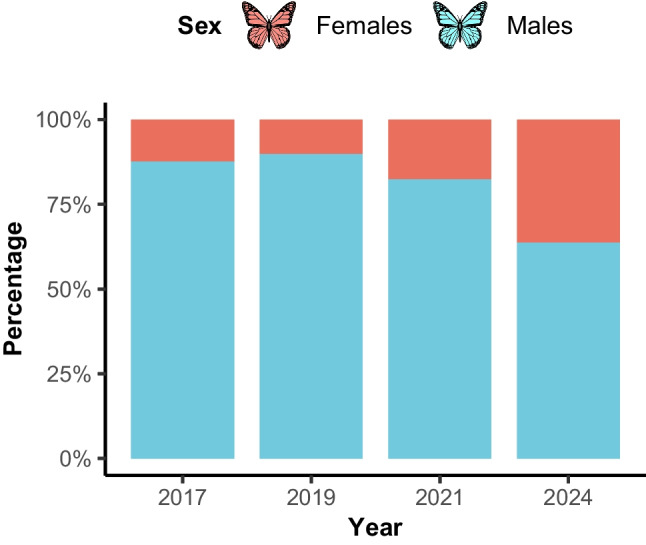
Sex ratio (percentage of males and females of *Baronia brevicornis*) across sampling years. Bars represent the relative proportion of males (blue/light) and females (orange/dark) captured each year. Data from 2018 were excluded due to extremely low and unbalanced sample size (n = 2; females only)

The sex ratio changed significantly across sampling years (χ^2^ = 204.47, df = 3, p < 0.0001; Pearson's chi-squared test; Fig. 1). However, the proportion of males was greater than females.

**Fig. 2:**
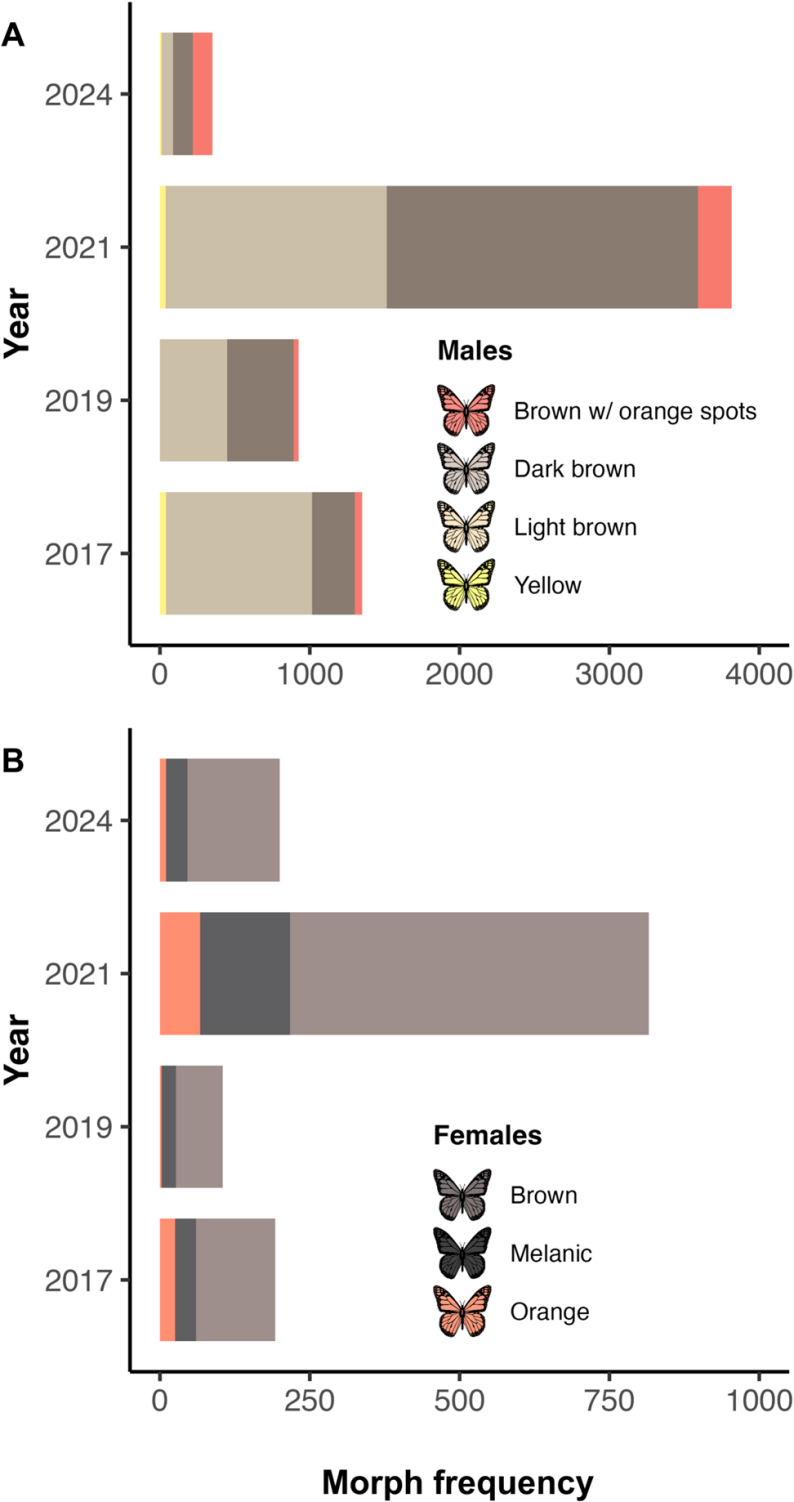
Frequency of male and female morphs of *Baronia brevicornis* observed in 2017, 2019, 2021, and 2024. A panel: male morphs. B panel: female morphs. The year 2018 was excluded due to the very limited number of observations (n = 2; brown female morph only)

The distribution of male morphs varied significantly across years (Fisher’s exact test with Monte Carlo simulation, B = 10,000, p < 0.001; Fig. 2A). The light brown morph decreased from 72.2% in 2017 to 22.0% in 2024, whereas the dark brown morph increased from 21.0% to 37.7% over the same period. The brown with orange spots morph showed a sharp increase in 2024, reaching 37.4%, after remaining below 6% in previous years. The yellow morph was consistently the least frequent, ranging from 0% to 3.1% across all years. Female morph frequencies also differed significantly among years (Fisher’s exact test with Monte Carlo simulation, B = 10,000, p < 0.0005; Fig. 2B). The brown morph was the most abundant throughout the study, representing 68.8% to 77.0% of females, depending on the year. The melanic morph fluctuated between 18.0% and 22.9%, while the orange morph remained the least common, ranging from 2.9% in 2019 to 13%.

**Fig. 3:**
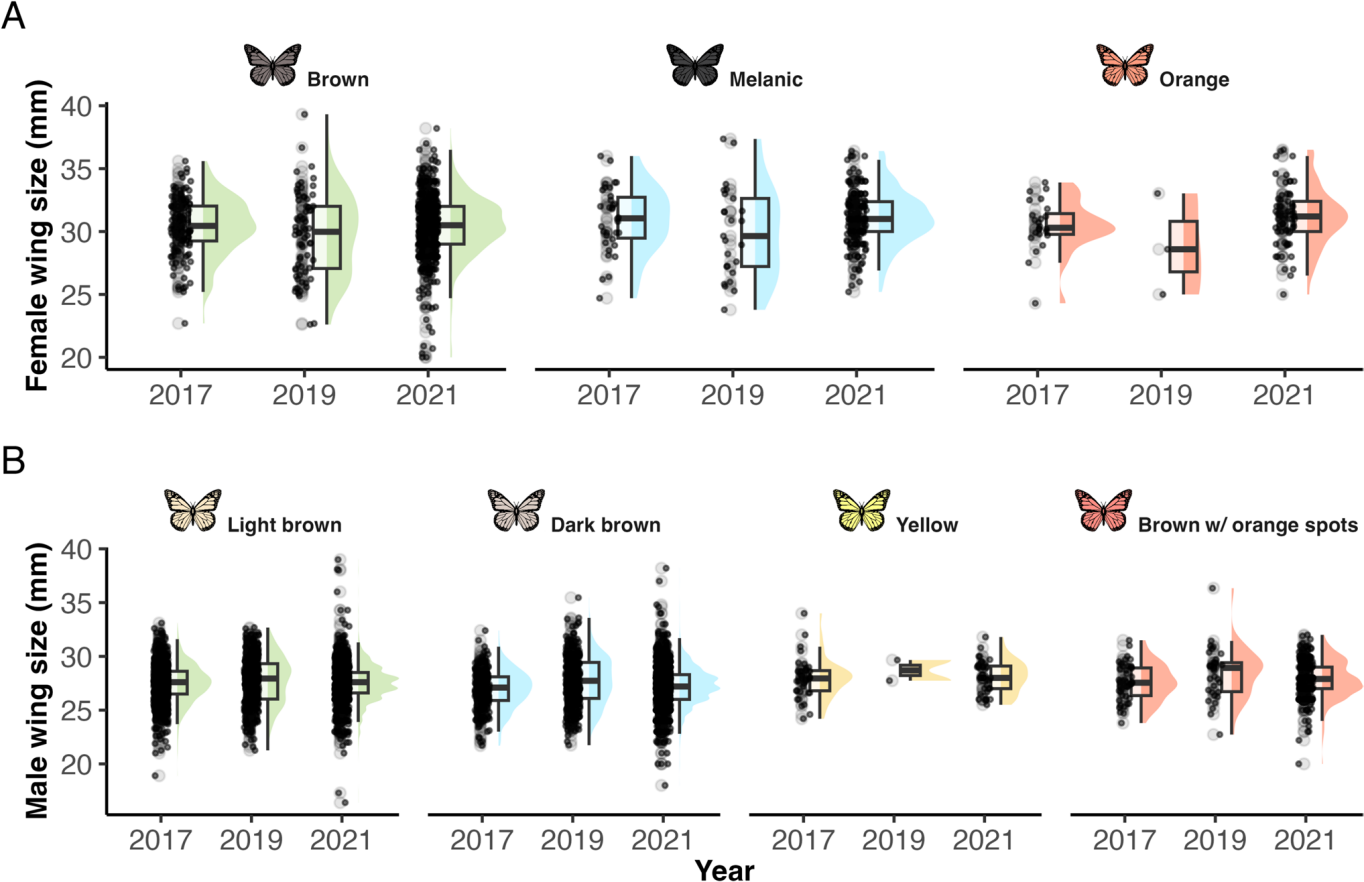
Wing size variation (mm) in A: females and B: males from *Baronia brevicornis* morphs across three years. Orange females and yellow males (the least common morphs) consistently showed the smallest wing sizes in all capture seasons. The years 2015, 2018, and 2024 were excluded due to the lack of wing size measurements

In females, wing size varied significantly across years (F_1,2_ = 4.83, p = 0.008) and among morphs (F_1,2_ = 5.08, p = 0.006; Fig. 3). In 2019, females had significantly smaller wings compared to other years (t_2_ = 2.84, p = 0.004, mean = 31.02 mm), whereas the largest wing size was recorded in 2021 (t_2_ = 0.51, p = 0.0006). Among female morphs, the melanic morph displayed the largest wings (t_2_ = 2.84, p = 0.004), while the orange morph had the smallest, although this difference was marginally non-significant (t_2_ = 1.84, p = 0.06). In males, wing size also differed significantly across years (F_1,2_ = 11.32, p < 0.0001) and among morphs (F_1,3_ = 14.54, p < 0.0001). The largest male wings were recorded in 2019 (t_2_ = 5.12, p < 0.0001, mean = 27.98 mm; Fig. 3). Among male morphs, the dark brown morph had the smallest wings (t_3_ = −3.77, p = 0.0002, mean = 27.42 mm).

### Abundance Trends and Detection Probability (N-mixture Model)

**Fig. 4:**
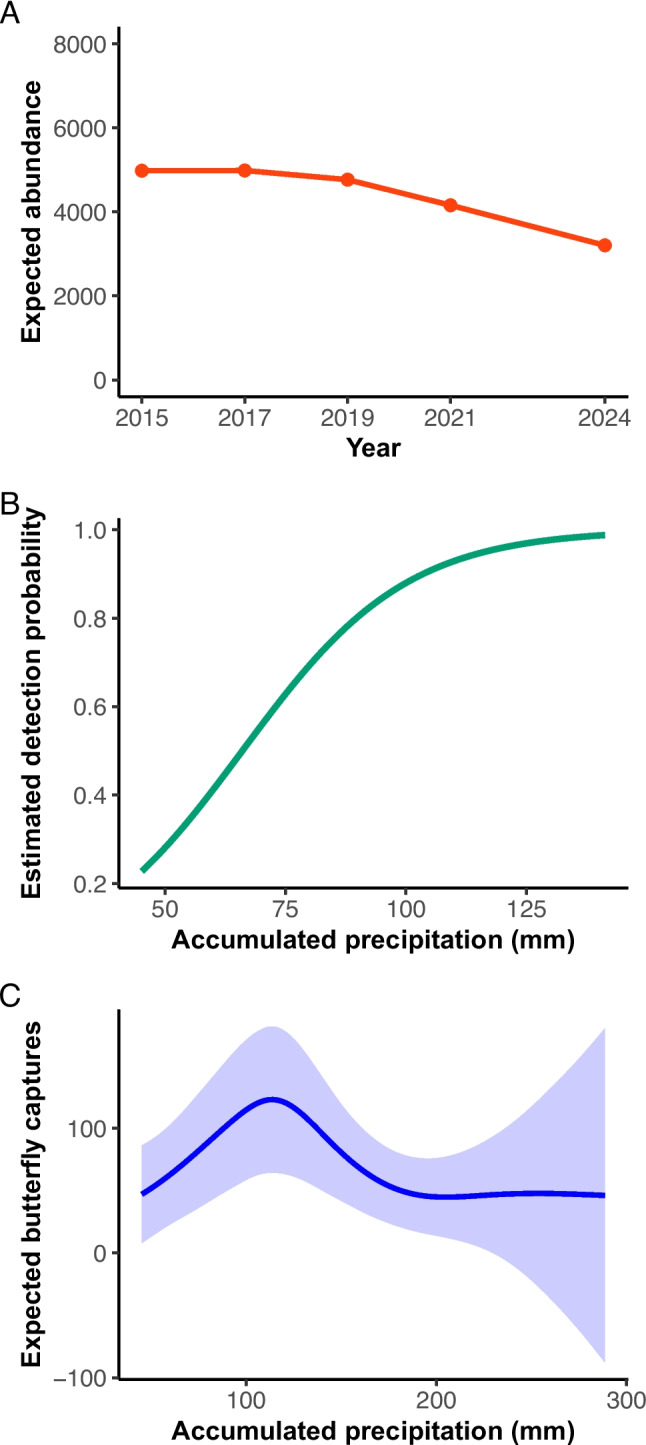
Expected abundance (**A**), detection probability (**B**) of *Baronia brevicornis* as a function of accumulated precipitation between 2015–2024 (data from 2018 were excluded due to the absence of repeated counts). Panel (**C**) shows the non-linear relationship between accumulated precipitation (mm) and the expected number of butterfly captures according to the GAM model. Shaded areas indicate 95% confidence intervals. An optimal precipitation range (~ 70–150 mm) was associated with the highest expected capture rates

The best supported N-mixture model (Negative Binomial distribution) revealed a significant decline in population size over time (β = −0.11, SE = 0.0335, z = −3.30, p < 0.001; Fig. 4A). Additionally, a higher proportion of males was associated with a greater abundance estimate (β = 0.09, SE = 0.04, z = 2.34, p = 0.02). Detection probability was not significantly influenced by maximum temperature (β = −0.02, SE = 0.03, z = −0.45, p = 0.64), but increased significantly with accumulated precipitation in the month preceding sampling (β = 0.06, SE = 0.02, z = 3.03, p = 0.0002; Fig. 4B). The dispersion parameter (β = 14.8, p = 0.66) was not significant, indicating that overdispersion was adequately accounted for by the model (see ESM_Figure 4 and ESM_Figure 5 for results of residual and collinearity analysis).

### Effect of Accumulated Precipitation and Maximum Temperature on Captures

The maximum temperature was not significantly associated with butterfly captures (p = 0.6) and was removed from the final model based on Akaike Information Criterion (AIC). The selected GAM retained year and accumulated precipitation as predictors, explaining ~ 73.4% of the deviance. Accumulated precipitation had a significant non-linear effect on the number of butterfly captures (χ^2^ = 11.34, edf = 3.34, p = 0.02), with an optimal range between ~ 70 and 150 mm, preceding the highest number of captures (Fig. 4C). This suggests that *B. brevicornis* emergence is strongly influenced by precipitation thresholds, where moderate rainfall creates favorable conditions for adult emergence, while lower or higher precipitation levels result in fewer captures. Although ‘*year’* was included as a predictor, its effect varied. Notably, in 2021, butterfly captures were significantly higher (Estimate = 1.19, p < 0.001), while in 2018, captures drastically decreased (Estimate = −6.16, p < 0.001), coinciding with field observations, where only two adult butterflies were recorded on the first day of the flight season, with no further individuals detected.

**Fig. 5:**
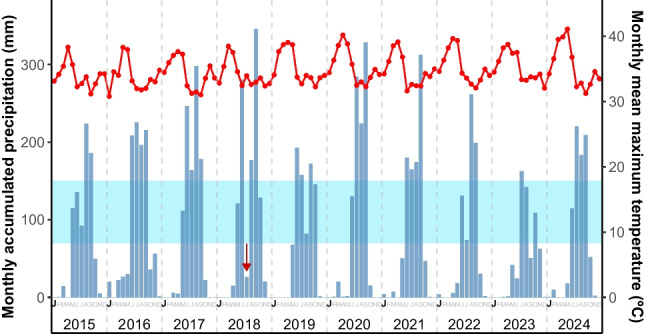
Monthly accumulated precipitation (blue bars) and monthly mean maximum temperature (red line) in the study area from 2015 to 2024. The shaded area indicates the optimal precipitation range (~ 70–150 mm) identified by the GAM as associated with the highest probability of adult butterfly captures. The red arrow highlights that in July 2018, rainfall did not fall within the threshold range, whereas in June, precipitation was abundant

For comparative purposes, Fig. 5 shows monthly accumulated precipitation and mean maximum temperature during the study period, highlighting in blue the optimal precipitation range identified by the GAM. This visualization allows placing the optimal range in the context of the interannual climatic variability experienced at the study site.

### Duration of Pupal Diapause

**Fig. 6:**
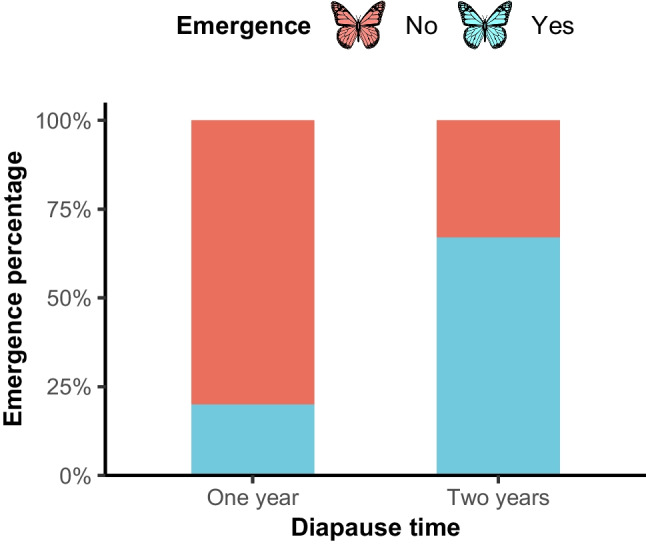
Percentage of adult emergence in *Baronia brevicornis* after one or two years of pupal diapause under moist conditions. Blue bars: successful emergence; orange bars: no emergence

During the first year of diapause, 80% of pupae successfully emerged following substrate moistening. In contrast, emergence markedly declined to 33% after a second year of diapause (χ^2^ = 6.89, df = 2, p = 0.03; Fig. 6), suggesting that extended diapause may negatively affect the likelihood of successful emergence.

## Discussion

The results reveal that *Baronia brevicornis* populations were consistently male biased across years, with significant interannual variation in morph frequencies and body size. We also detected a declining trend in butterfly abundance, with higher detection probabilities following periods of increased rainfall. Maximum temperature did not significantly influence captures, whereas accumulated precipitation within an optimal range (~ 70–150 mm) prior to the reproductive season was strongly associated with the highest probability of capture. Additionally, the diapause experiment showed that *B. brevicornis* can prolong pupal diapause for at least two years, although emergence success markedly diminished during the second.

Our findings reveal a persistent male-biased sex ratio across multiple years, aligning with earlier reports by Galicia-Mendoza et al. (2021). This could be explained by two non-exclusive mechanisms: (1) higher female mortality after emergence and mating, or (2) female dispersal from emergence sites to oviposition areas, with males remaining nearby to increase mating opportunities. Although the primary sex ratio at pupal emergence is balanced (Galicia-Mendoza et al. 2021), the operational sex ratio at the capture sites favors males. Disentangling whether this sex difference arises from differential survival or behavioral segregation is essential. Similar phenomena have been documented in other insects, such as *Hetaerina americana*, where females segregate spatially to avoid harassment and only temporarily aggregate at feeding or roosting sites (Grether & Donaldson 2007). In *B. brevicornis*, females may similarly leave the emergence area after mating and oviposition.

Morph frequencies varied significantly across years for both sexes, with some morphs showing marked increases or declines, while others remain consistently rare. The persistence of certain morphs, such as the yellow in males and orange in females, despite their low frequency, suggests a possible genetic basis for morph expression (Leimar 2009). This pattern mirrors cases like *Erythrura gouldiae*, where stable morph proportions are maintained through genetic polymorphism despite shifts in dominance (Pryke & Griffith 2009). Such stability, coupled with interannual variation, points to a complex interplay between environmental influences, genetic factors and possibly assortative mating (Tregenza & Wedell 2000) in shaping morph distribution in *B. brevicornis*.

Wing size also varied among morphs and years, with smaller individuals recorded in 2019 and larger ones in 2021. As size in insects is often a condition-dependent trait (McConnell & Judge 2018; Gergely & Tökölyi 2023), these differences may reflect variation in larval resource availability or environmental conditions. Future research should focus on the environmental influences on morph-specific coloration and size traits. Additionally, it should be investigated whether the morphs exhibit redundant signals of condition, or whether each morph conveys distinct indicators of condition (e.g., immune response, oxidative stress or predation avoidance).

The N-mixture model revealed that a higher proportion of males was associated with increased abundance estimates, which could reflect a demographic bias previously mentioned. From a conservation perspective, a consistently male-biased sex ratio could negatively impact reproductive potential and population resilience, especially under climate change scenarios (Bonal et al. 2015). This hypothesis warrants further research. In addition, the N-mixture model showed a declining abundance trend over time. While natural interannual fluctuations are common in butterfly populations and often driven by climatic variability (Pollard & Yates 1993), the consistent downward trajectory observed suggests that additional pressures (anthropogenic and/or environmental) may be contributing to this decline.

The GAM analysis revealed a non-linear relationship between accumulated precipitation and butterfly captures, with an optimal threshold range (~ 70–150 mm) preceding peak emergence. Values below or above this range reduced captures, indicating that emergence is sensitive not only to the amount of rain but also to its timing and persistence. Monthly totals provide a general climatic context, but early-season rainfall patterns are critical. Late onset, short but intense episodes, or long dry intervals may fail to sustain soil moisture long enough to trigger emergence and promote metabolic activation (Schwinning & Sala 2004; Hahn & Denlinger 2007). Conversely, excessive rainfall (despite increasing monthly totals) may cool the soil, delay pupal development, or reduce adult activity (McDermott Long et al. 2017). Intense rains during the flowering period of the host plant (*A. cochliacantha*) could also disrupt phenological synchrony, causing premature flower drop or damage to shoots, potentially reducing nectar availability and limiting emergence. The marked emergence decline in 2018 exemplifies this sensitivity: Rainfall surpassed the optimal threshold range in June but declined below it in July, conditions unlikely to sustain emergence. In contrast, the higher captures in 2021 and 2024 probably occurred in years when rainfall patterns stayed within the optimal range. Although maximum temperature did not influence captures in our models, it may affect other aspects of biology such as activity levels, mating behaviors or survival. These are factors beyond the scope of this study but warrant further research.

Precipitation functions as a critical environmental cue influencing the behavioral dynamics of *B. brevicornis*, with significant implications for its population dynamics (Tauber et al. 1998; Henry et al. 2022). Although limited research has directly examined the effects of moisture and temperature variations on diapause maintenance, termination, or adult flight behaviors, existing studies suggest that these factors play essential roles. For example, larval diapause initiation and termination in *Busseola fusca* depend on moisture availability (Okuda 1990, 1991), and in *Acacia* species, precipitation influences leaf production (Milton 1987). Excessive rainfall, however, can threaten ecological interactions such as pollinator-plant relationships by increasing mortality rates or reducing resource availability (Lawson & Rands 2019). Understanding how *B. brevicornis* pupae perceive optimal precipitation conditions for pupal emergence and how rainfall thresholds regulate adult presence in the field could reveal critical mechanisms driving population dynamics. Rainfall fluctuations may explain why *B. brevicornis*, akin to other insects with facultative diapause, is capable of extending its developmental arrest during periods of inadequate rainfall. Such plasticity may be an adaptive strategy to navigate the unpredictability characteristic of subtropical environments where this butterfly occurs. Our findings corroborate experimental evidence demonstrating that *B. brevicornis* can prolong diapause but also indicates a cost: emergence success dropped from 80% after one year to 33% after two years of diapause. The decline in pupal emergence following prolonged diapause aligns with the observed decrease in numbers after low precipitation in 2018, suggesting increased pupal mortality associated with extended diapause periods. This pattern parallels findings in species like *Thaumetopoea pityocampa* and *T. wilkinsoni*, where extended diapause correlates with resource depletion (Salman et al. 2019; Saunders 2000). Despite the metabolic suppression characteristic of diapause, environmental factors such as temperature fluctuations can stimulate resource consumption, particularly in warmer climates, impacting overall metabolic activity (Hahn & Denlinger 2007). The energy reserves accumulated post-diapause are crucial for developmental transitions, including metamorphosis, as well as for survival and dispersal activities (Hahn & Denlinger 2007). Investigating the physiological and ecological costs associated with prolonged pupation and diapause in *B. brevicornis* represents a promising direction for future research. Interestingly, *B. brevicornis* exhibits a high level of heterozygosity (~ 0.58%), which may seem contradictory given its endangered status (Marino et al. 2023). However, its vulnerability warrants further investigation, especially considering the role of cumulative precipitation and the potential for diapause extension within the same year (rather than into subsequent years) when environmental conditions are favorable. Moreover, extending diapause beyond one year could entail substantial energetic and survival costs, particularly if regional rainfall patterns remain consistently low. As a panchronic species often inhabiting highly specific environments, *B. brevicornis* is likely sensitive to environmental fluctuations. Although these findings may not be universally applicable to all panchronic species, they highlight the importance of evaluating how climate variability influences their population persistence and resilience.

Finally, we recommend future research to investigate the influence of non-climatic variables on the population decline, morph frequency, and diapause strategies of *B. brevicornis*. Although tropical dry forests are biodiversity-rich ecosystems (Rzedowski 1991), they are subject to ongoing anthropogenic pressures, primarily land-use change, deforestation, and cattle ranching (Mesa-Sierra et al. 2022). These activities modify vegetation structure, soil properties, and reduce overall habitat extent (Lozano-García et al. 2021), yet their impacts on *B. brevicornis* populations and associated plant communities remain largely unexamined. Furthermore, the species is preyed upon by several predators, including the beetle *Calosoma angulatum* (Covarrubias-Camarillo et al., 2015), lizards (*Aspidoscelis communis* and *A. deppeii*; Legal et al. 2015), and birds (*Tyrannus vociferans, Myiarchus tyrannulus, and Geococcyx velox*; Méndez-Muñiz 2025, unpublished data). However, it remains unclear whether these predators exert phenological or seasonal pressures on *B. brevicornis*. Studies should aim to elucidate the roles of these biotic interactions and other potential factors driving population decline and shifts in morph frequencies to develop a comprehensive understanding.

In conclusion, we found evidence of phenological plasticity in both male and female morphs, as well as in wing size, with some morphs exhibiting greater plasticity than others. The adaptive capacity of *B. brevicornis* (particularly its ability to extend diapause) appears to enhance its resilience to climatic variability. This strategy may help avoid emergence during periods of insufficient rainfall, thereby increasing the likelihood of adult survival. Such plasticity could play a key role in buffering populations against the effects of climate change in subtropical environments. However, these strategies likely operate within limits, with an optimal threshold range of accumulated precipitation required for successful emergence from diapause. Understanding these limits, along with the effects of habitat change and biotic interactions, will be crucial for developing conservation strategies for this and other panchronic species in subtropical environments.

## Supplementary Information

Below is the link to the electronic supplementary material.

ESM 1 (DOCX 2.70 MB)

ESM 2 (XLSX 397 KB)

## Data Availability

The datasets generated and analyzed during the current study are available as supplementary material (ESM_2_data).
