# Supplementary material for: Precipitation and Interannual Variability Shape the Phenology and Abundance of the Endangered Butterfly Baronia brevicornis
Source: Neotrop Entomol. 2025 Dec 23;54(1):131. doi: 10.1007/s13744-025-01325-y (PMC12727813; doi:10.1007/s13744-025-01325-y)
Supplement: Supplementary file 1 — (DOCX 2.70 MB) [file 13744_2025_1325_MOESM1_ESM.docx]

**Precipitation and Interannual Variability Shape the Phenology and Abundance of the Endangered Butterfly *Baronia brevicornis***

**Running title: Phenology and abundance of *Baronia brevicornis***

Yesenia Alvarado-Campos^1^, Gloria Ruiz-Guzmán^1^, Carlos A. Anaya Merchant^1^, Elaine M. Méndez Muñiz^1,2^ & Jorge Contreras-Garduño^1*^

^1^ Escuela Nacional de Estudios Superiores, Unidad Morelia, UNAM, Morelia, Michoacán, México.

^2^ Posgrado en Ciencias Biológicas, UNAM, México.

***Correspondence:** Jorge Contreras-Garduño, Escuela Nacional de Estudios Superiores, Unidad Morelia, UNAM, Antigua Carretera a Pátzcuaro No.8701, Col. Ex-Hacienda San José de la Huerta. CP 58190, Morelia, Michoacán, México. E-mail: [jcg@enesmorelia.unam.mx](mailto:jcg@enesmorelia.unam.mx)

**KEYWORDS**

Panchronic species; Diapause; Polymorphism; Climate change; Endemic; Plasticity; Precipitation

ESM_Figure 1 A female brown morph of *Baronia brevicornis* perched on *Acacia cochliacantha* (the blue spots on the wing are due to marking)


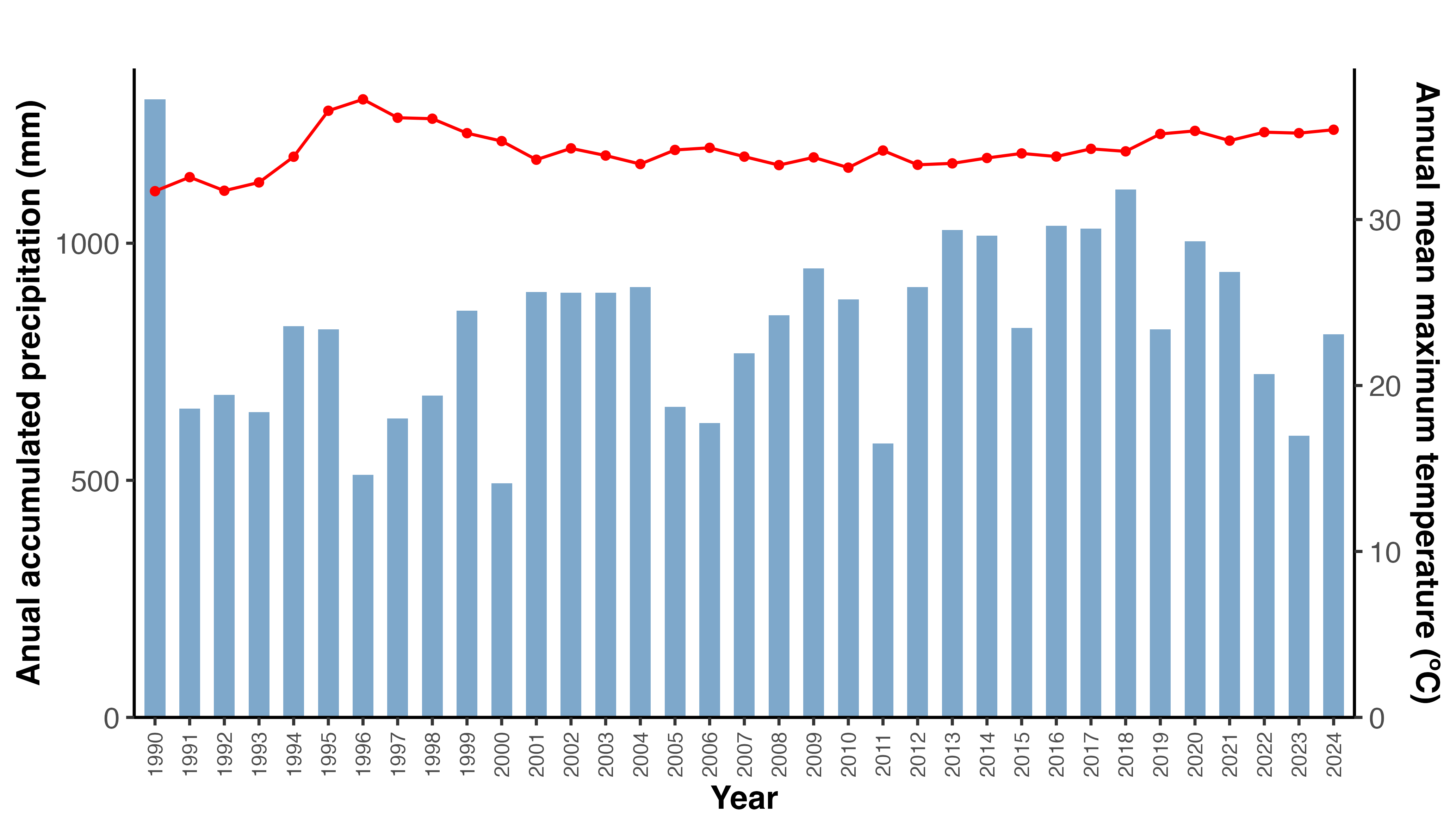


ESM_Figure 2 Historical data of annual precipitation (blue bars) and average annual maximum temperature (red line) of the study site from 1990 to 2024


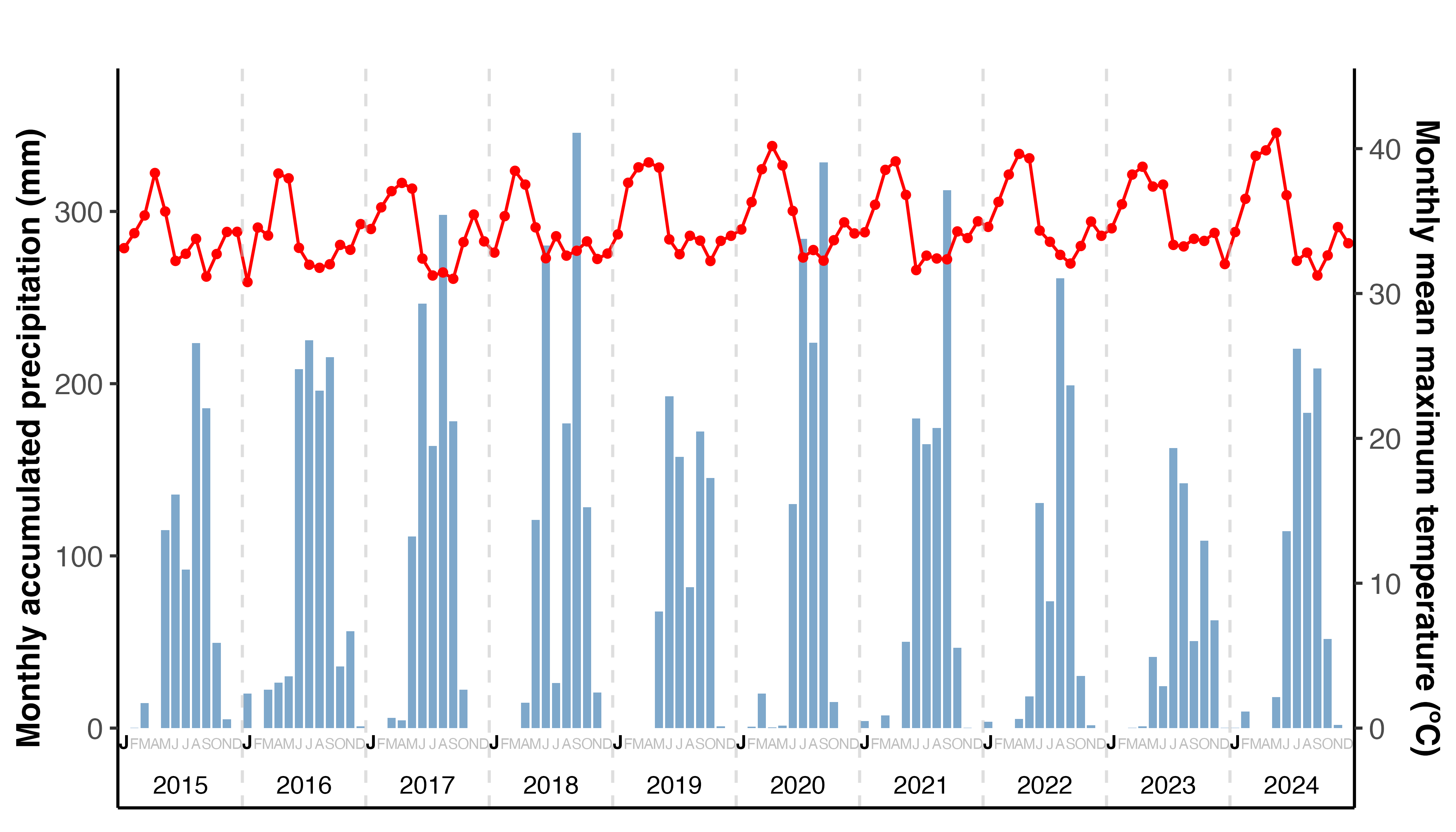


ESM_Figure 3 Monthly accumulated precipitation (blue bars) and monthly mean maximum temperature (red line) in the study area from 2015 to 2024


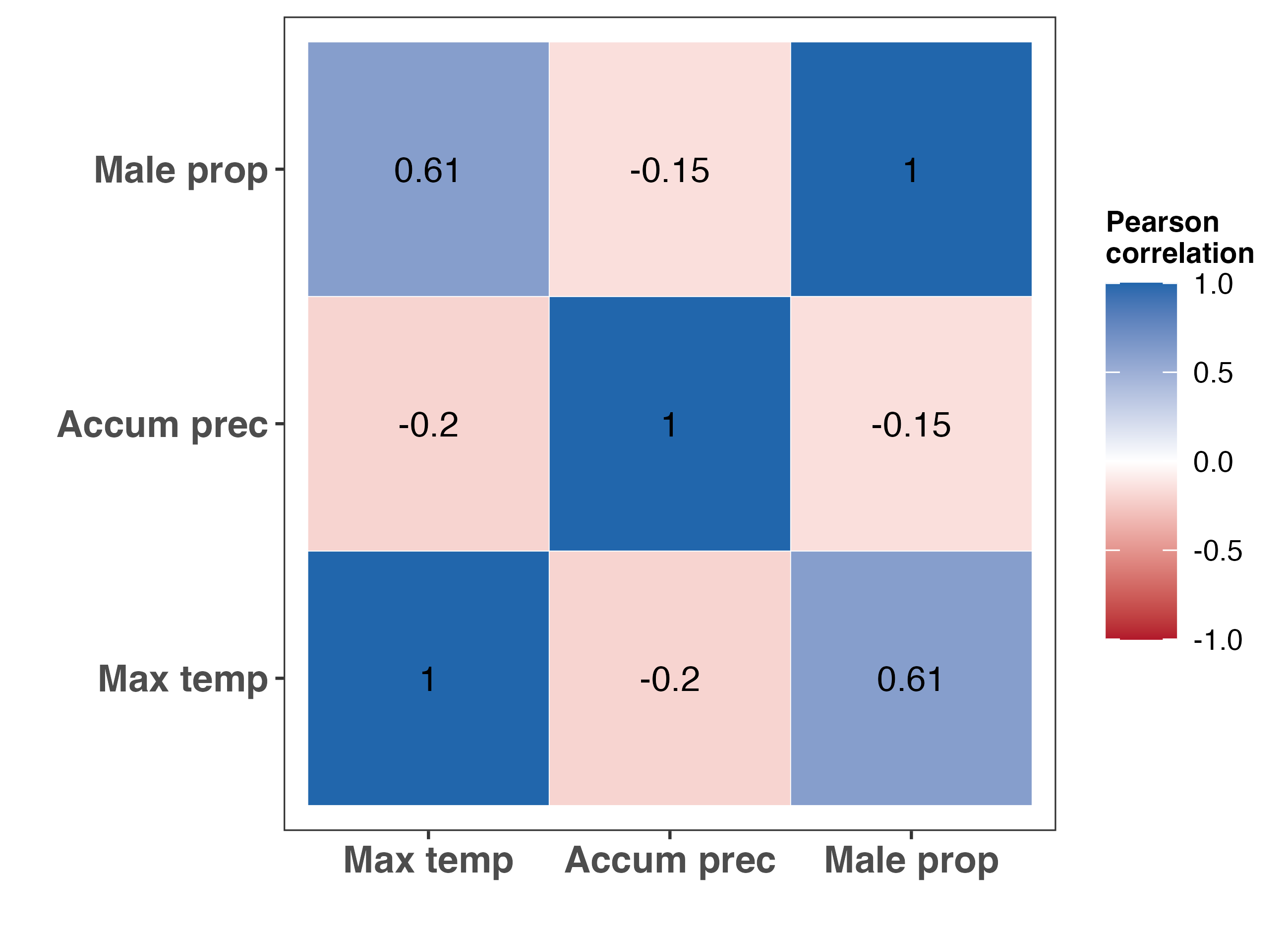


ESM_Figure 4 Pearson correlation matrix among the covariates used in the N-mixture model. All pairwise correlations were below 0.7, indicating low collinearity among predictors (Dorman et al., 2013)


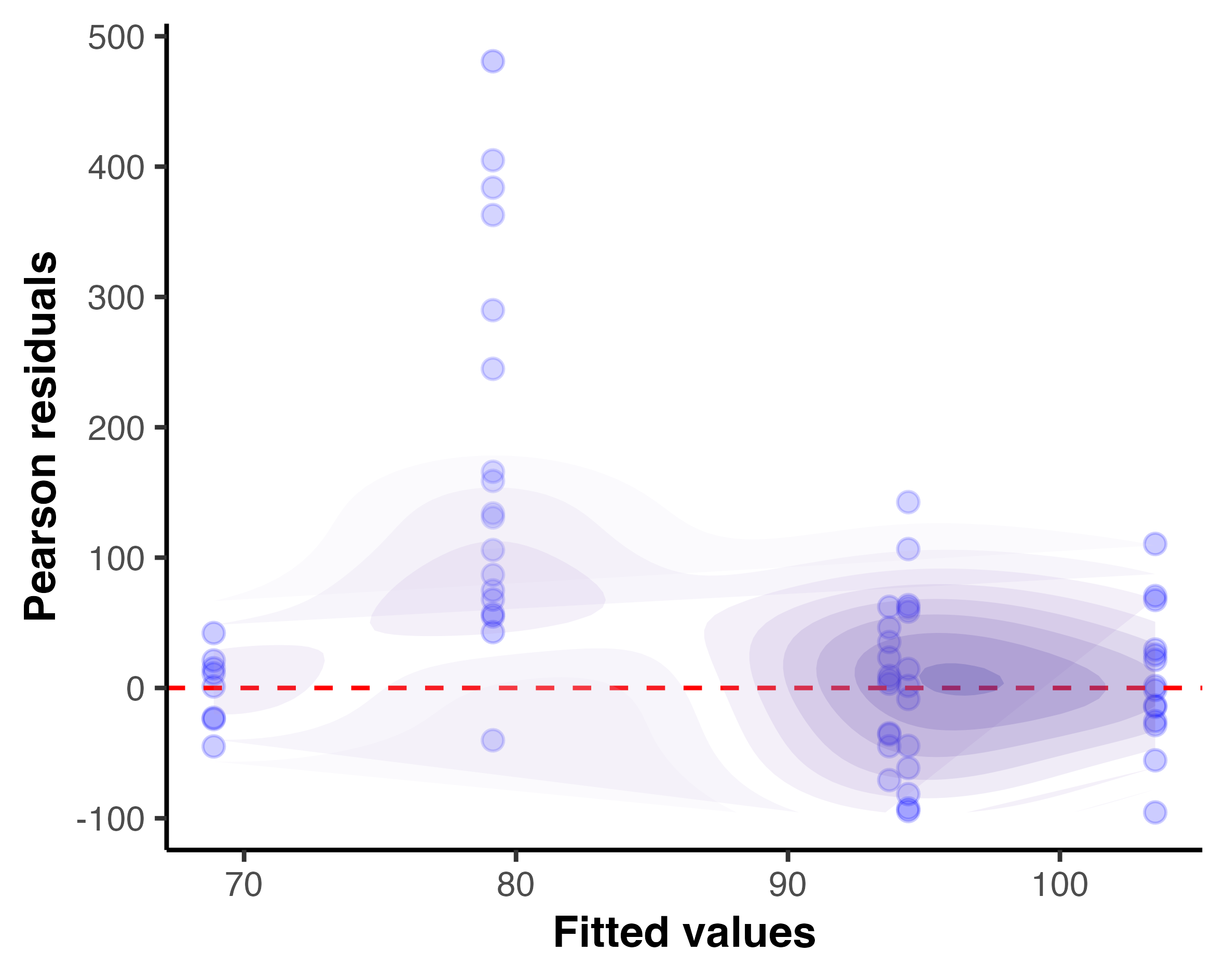


ESM_Figure 5 Pearson residuals plotted against fitted values for the N-mixture model. Residuals show no clear patterns, supporting the adequacy of the model fit

**References**

Dormann, C. F., Elith, J., Bacher, S., Buchmann, C., Carl, G., Carré, G., ... & Lautenbach, S. (2013). Collinearity: a review of methods to deal with it and a simulation study evaluating their performance. Ecography, 36(1), 27-46.
